# Aminoacylation‐defective bi‐allelic mutations in human EPRS1 associated with psychomotor developmental delay, epilepsy, and deafness

**DOI:** 10.1111/cge.14269

**Published:** 2022-12-01

**Authors:** Danni Jin, Sheree A. Wek, Ricardo A. Cordova, Ronald C. Wek, Didier Lacombe, Vincent Michaud, Karin Musier‐Forsyth

**Affiliations:** ^1^ Department of Chemistry and Biochemistry Center for RNA Biology, Ohio State University Columbus Ohio USA; ^2^ Department of Biochemistry and Molecular Biology Indiana University School of Medicine Indianapolis Indiana USA; ^3^ Department of Medical Genetics University Hospital of Bordeaux Bordeaux France; ^4^ INSERM U1211, Rare Diseases, Genetics and Metabolism University of Bordeaux Bordeaux France

**Keywords:** aminoacylation, aminoacyl‐tRNA synthetase, EPRS1, exome sequencing, integrated stress response, leukodystrophy, psychomotor developmental delay, tRNA

## Abstract

Aminoacyl‐tRNA synthetases are enzymes that ensure accurate protein synthesis. Variants of the dual‐functional cytoplasmic human glutamyl‐prolyl‐tRNA synthetase, EPRS1, have been associated with leukodystrophy, diabetes and bone disease. Here, we report compound heterozygous variants in *EPRS1* in a 4‐year‐old female patient presenting with psychomotor developmental delay, seizures and deafness. Functional studies of these two missense mutations support major defects in enzymatic function in vitro and contributed to confirmation of the diagnosis.

## Graphical abstract

We report compound heterozygous variants in a bifunctional aminoacyl‐tRNA synthetase, EPRS1, in a 4‐year‐old female patient presenting with psychomotor developmental delay, seizures and deafness. Functional studies of these two missense mutations support major defects in enzymatic function in vitro and contributed to confirmation of the diagnosis.

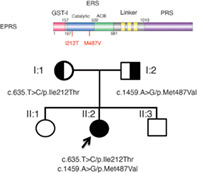

## 1 INTRODUCTION

Aminoacyl‐tRNA synthetases (ARSs) are essential enzymes that catalyze the charging of amino acids onto their cognate tRNAs using a two‐step aminoacylation reaction. To date, disease‐associated mutations have been reported in 18 out of the 20 cytoplasmic ARSs^1^ and all 19 of the nuclear‐encoded mitochondrial ARSs.^2^ Whereas a broad range of disorders are associated with ARS mutations, the most common afflict the brain and neuromuscular systems.^1^, ^2^

The human *EPRS1* gene encodes cytoplasmic glutamyl‐prolyl‐tRNA synthetase (EPRS1), a bifunctional ARS that catalyzes the charging of both glutamic acid and proline to their corresponding tRNAs. In human EPRS1, a glutamyl‐tRNA synthetase (ERS) domain and a prolyl‐tRNA synthetase (PRS) domain are connected by a highly flexible linker region. Bi‐allelic point mutations in *EPRS1* have been discovered in patients with hypomyelinating leukodystrophy,^3^ diabetes and bone diseases.^4^

In this study, we report novel compound heterozygous point mutations in the ERS region of the *EPRS1* gene of a 4‐year‐old patient with psychomotor retardation, epilepsy, and deafness. We biochemically characterized the impact of these mutations on the structure and function of the recombinant ERS protein variants in vitro and performed initial studies on patient‐derived fibroblasts. Despite a severe defect in tRNA aminoacylation kinetics in vitro, no cellular growth defect was observed and the integrated stress response (ISR) commonly triggered by global defects in protein synthesis, was not upregulated. This likely reflects the known tissue‐specific differences in the sensitivity of cells to aminoacylation defects, with neurological tissues displaying high sensitivity.^5^, ^6^

## 2 METHODS

Library preparation, exome capture, sequencing and data analysis were performed by IntegraGen SA (Evry, France) using Twist Bioscience in‐solution enrichment methodology (Agilent, Santa Clara, California), followed by paired‐end 100 base massively parallel sequencing on Illumina NovaSeq (Illumina, San Diego, California). Additional details are found in Supporting Information.

Recombinant ERS proteins (WT and mutants) encoding N‐terminal small ubiquitin‐like modifier (SUMO) and maltose binding protein (MBP) tags followed by EPRS1 residues 1‐749 were expressed in *Escherichia coli* and purified as previously described.^4^ Charging of tRNA^Glu^ was measured by aminoacylation assays.^4^ Quantitative electromobility gel shift assays (EMSAs) were used to assess ERS affinity for tRNA^Glu^. Protein conformation was analyzed using circular dichroism (CD) spectroscopy and limited protease digestion assays.^4^ Detailed protocols are found in Supporting Information.

Patient‐derived fibroblasts were obtained from an arm skin biopsy and control fibroblasts were from a 13‐year‐old healthy boy. DNA sequencing was carried out to confirm the cell lines had compound heterozygote mutations for *EPRS1*. Detailed protocols for immunoblotting, qRT‐PCR, cell viability and protein synthesis measurements are found in Supporting Information.

## 3 RESULTS

### 3.1 Clinical results

**FIGURE 1:**
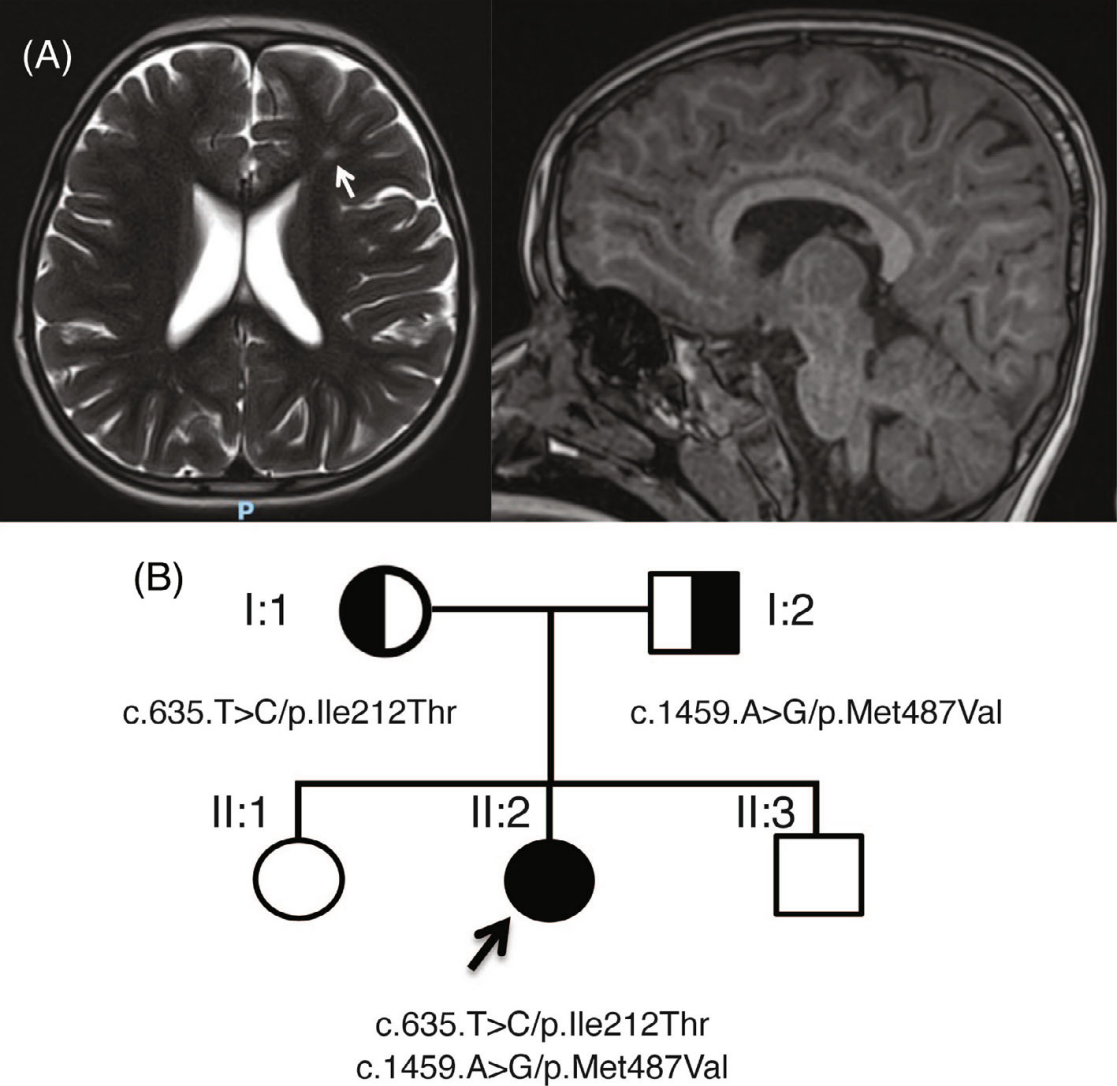
Patient brain MRI images and family pedigree. (A) Brain MRI images of patient II:2 showing hypersignal of left lateral periventricular white matter (white arrow) and a certain degree of cortical atrophy (*left*, Axial T2 Blade sequence; *right*, Sagittal T1 sequence). (B) Family pedigree showing affected patient II:2 bearing two pathogenic *EPRS1* variants (compound heterozygous). Parents I:1 and I:2 are nonaffected and each bear one heterozygous pathogenic *EPRS1* variant. [Colour figure can be viewed at wileyonlinelibrary.com]

Patient II:2 was born at term from healthy nonconsanguineous parents after an uneventful pregnancy. At birth she weighed 2.59 kg (−1 standard deviation, SD) measured 47 cm in length (−1 SD) and had a 32 cm head circumference (−1 SD). She developed West syndrome at 9 months and was treated with three different lines of anti‐epileptic drugs and put on a ketogenic diet. At her last examination she was 3 years old and weighed 15.5 kg (median, M), measured 98 cm (M) and had a head circumference of 48 cm (−1.5 SD). She presented with psychomotor delay as she was able to sit and crawl but had not acquired independent walking. She has no verbal speech. Examination of the patient found plagiocephaly, deep set eyes, and peripheric hypertonia. She is in normal kindergarten school but needs assistance and is undergoing speech, psychomotor and physical therapy. She had a normal ophthalmologic examination, a normal computed tomography scan of the brain (brain tomodensitometry) and a normal renal ultrasound. Brain magnetic resonance imaging found a hyperintensity of periventricular white matter and cortical atrophy (Figure 1A). An audiogram performed found bilateral moderate deafness (60–80 dB) and her electroencephalography was abnormal. Genetic screening found a normal array CGH and normal epilepsy NGS panel. Whole‐exome sequencing found two variants, resulting in a compound heterozygous variant *EPRS1* gene (Figure S1). Combined Annotation Dependent Depletion (CADD) phred scores are used to predict the deleteriousness of single nucleotide variants with a score above 20 indicating a probable pathogenicity of the variant. As illustrated in the pedigree shown in Figure 1B, the first variant NM_004446.3:c.1459A > G/p.Met487Val was inherited from the patient's father and has a CADD phred score of 25. The second variant NM_004446.3:c.635 T > C/p.Ile212Thr was inherited from her mother and has a CADD phred score of 28.20. Both variants were absent from the gnomAD database and were classified as pathogenic, as described in the Supporting Information.

### 3.2 Biochemical results

**FIGURE 2:**
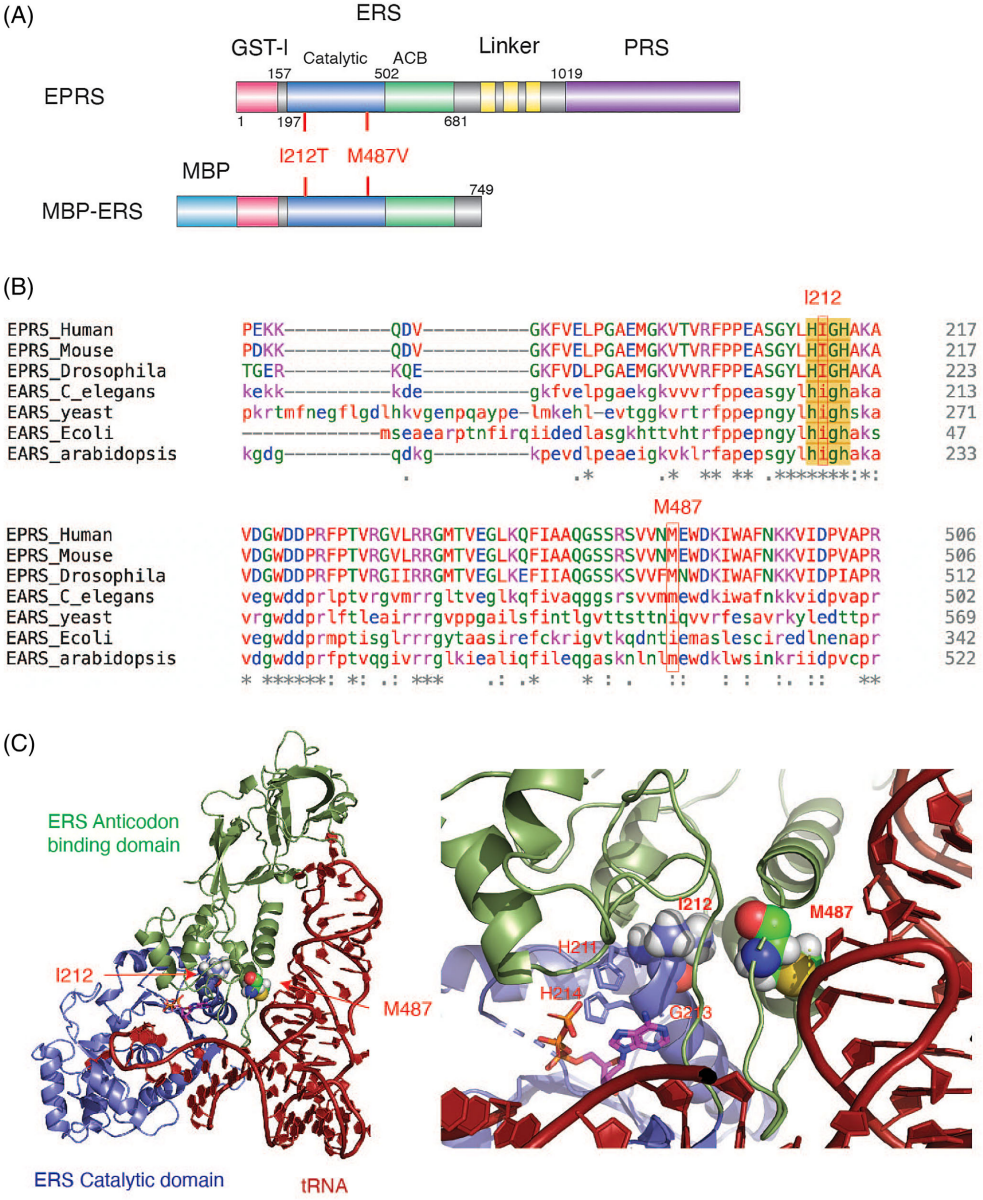
Human ERS structural models and location of disease‐associated mutations. (A) Schematic of full‐length human EPRS and recombinant MBP‐tagged ERS protein. ACB is anticodon binding domain. (B) Sequence alignment of ERS across multiple species. The HIGH Class I signature sequence containing I212 is highlighted in yellow and the semi‐conserved M487 residue is boxed. Amino acids are in upper case for EPRS and lower case for ERS sequences. (C) Homology model of human ERS‐tRNA complex based on *E. coli* GlnRS. A zoomed‐in view of M487‐D arm region is shown on the right with residues of the HIGH sequence indicated. [Colour figure can be viewed at wileyonlinelibrary.com]

Positions I212 and M487 of human EPRS1 are both located in the catalytic domain of ERS (Figure 2A). The I212 residue is strictly conserved among species (Figure 2B) and is within the HIGH signature sequence of Class I ARSs, which is critical for ATP binding and amino acid activation.^7^ The M487 residue is conserved among animal and plant species, while in yeast and bacteria it is replaced with an isoleucine residue (Figure 2B). Based on a model of the ERS‐tRNA complex,^4^ the M487 residue is near the ERS‐tRNA interface with its side chain proximal to the tRNA phosphate backbone (Figure 2C).

**FIGURE 3:**
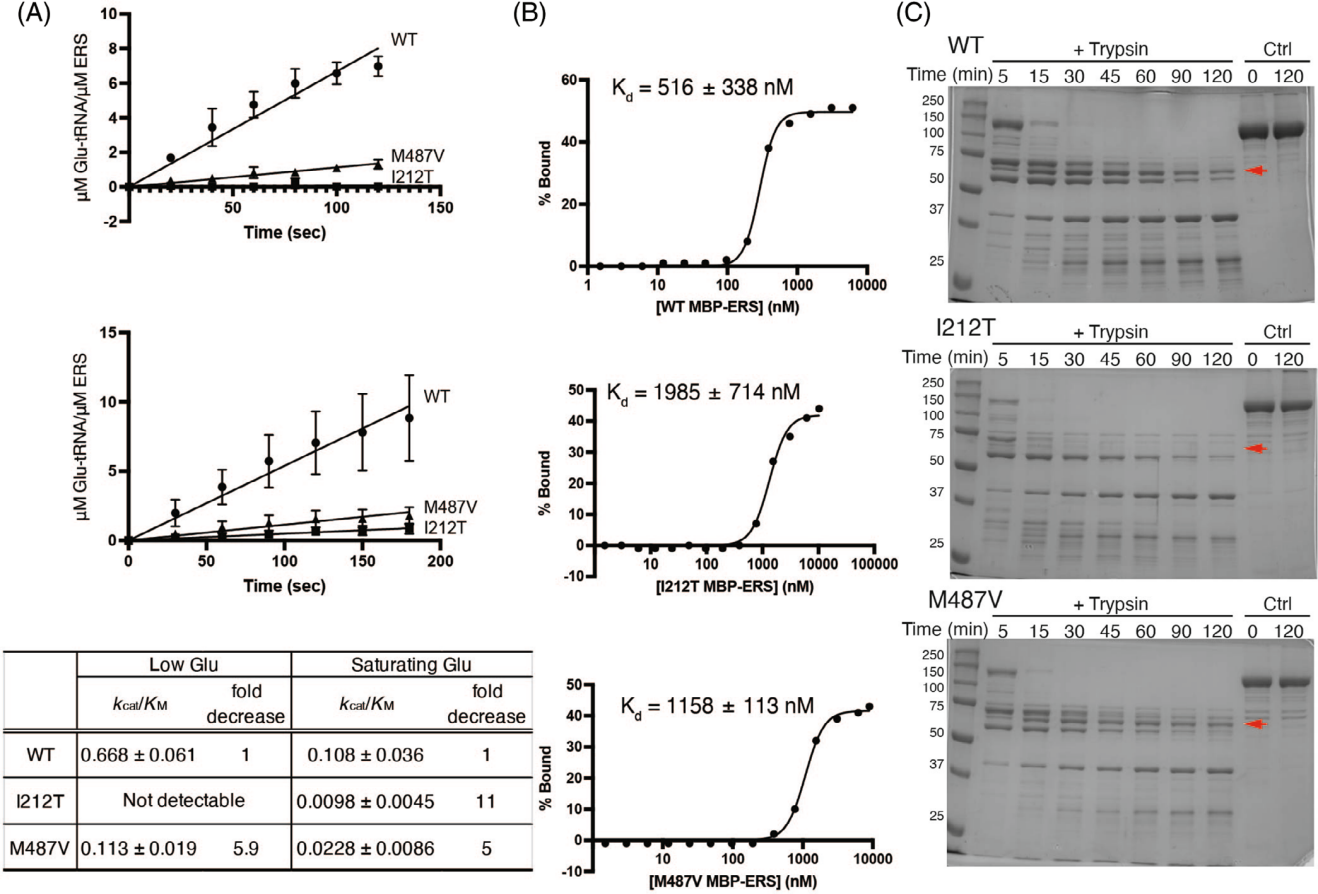
Aminoacylation activity, tRNA^Glu^ affinity, and limited trypsin digestion of WT and mutant MBP‐ERS. (A) *Top*: Aminoacylation assays performed with 4 nM (WT) or 10 nM (mutants) ERS, 0.1 μM tRNA^Glu^, and 20 μM [^3^H]‐glutamic acid. Middle: Aminoacylation assays performed with 10 nM ERS, 0.5 μM tRNA^Glu^, 1 mM glutamic acid and [^32^P]‐tRNA^Glu^. Each experiment was performed at least in triplicate and linear fits of the data are shown. *Bottom*: Catalytic efficiency estimated based on initial velocities. (B) tRNA^Glu^ binding affinity determined by EMSAs. Purified proteins were titrated into 10 nM [^32^P]‐tRNA^Glu^: WT MBP‐ERS (top), I212T MBP‐ERS (middle), and M487V MBP‐ERS (bottom). Each assay was performed in triplicate and one representative trial is shown. Dissociation constants (K_d_) were derived by fitting the binding curves to Hill equation. (C) Limited tryptic digests. WT (top), I212T (middle) and M487V (bottom) MBP‐ERS proteins were subjected to trypsin digestion for up to 2 h. Digested protein samples were separated by denaturing SDS‐polyacrylamide gel electrophoresis and visualized by Coomassie Brilliant Blue staining. A representative image for each protein variant is shown. Two independent trials were performed for each protein, and red arrows indicate the digested fragment with different stability among MBP‐ERS variants. [Colour figure can be viewed at wileyonlinelibrary.com]

Recombinant MBP‐ERS proteins carrying disease‐associated point mutations were expressed in *E. coli* and purified (Figure S2A). The aminoacylation activity of WT and mutant MBP‐ERS was first measured in the presence of [^3^H]‐Glu. Catalytic efficiency *k*_cat_/*K*_M_ was estimated based on the initial rate of Glu‐tRNA^Glu^ formation (Figure 3A, top). M487V mutation resulted in a 5.9‐fold decrease in catalytic efficiency relative to WT, whereas the catalytic activity of the I212T variant was not detectable. Commercially available [^3^H]‐labeled amino acids have low‐specific activities, preventing the use of saturating concentrations.^8^ To circumvent this caveat, we performed aminoacylation assays using 3′‐[^32^P]‐labeled tRNA and 1 mM of unlabeled Glu (Figure 3A, bottom).^9^, ^10^ Relative to WT ERS, the *k*_cat_/*K*_M_ values determined for I212T and M487V variants were reduced 11‐ and 5‐fold, respectively. Both assays suggest that the I212T mutation has a more dramatic effect on ERS function than the M487V mutation.

We performed quantitative EMSAs to test tRNA^Glu^ binding affinity. A dissociation constant of 516 nM was measured for WT MBP‐ERS, whereas the I212T and M487V variants bound tRNA with 4‐ and 2‐fold reduced affinity (*K*_d_ of ~2 μM and ~1.2 μM, respectively) (Figures S2B and 3B). The reduced affinity for M487V is consistent with the predicted close proximity of M487 to the tRNA backbone (Figure 2B). The larger effect of I212T, which is located ~8 Å from M487, is likely a more indirect effect.

CD spectroscopy studies showed that both mutant proteins folded into a primarily alpha‐helical structure similar to the WT protein (Figure S2C, top) and thermal melting studies showed that the apparent melting temperature (T_m_) was not affected by the mutations (Figure S2C). A limited proteolysis assay used to probe more subtle conformational differences showed that WT and M487V MBP‐ERS proteins displayed similar trypsin digestion patterns (Figure 3C). For the I212T variant, the ~60 kD fragment (red arrow) was much more rapidly degraded, suggesting that this mutation alters the conformation of MBP‐ERS. Computational approaches were also used to predict the effect of the amino acid substitutions on EPRS1 protein structure. As described in Supporting Information: Methods and Results, these predictions are consistent with altered protein flexibility and stability, especially for the I212T variant.

### 3.3 Cell‐based results

Disruptions in protein homeostasis that arise in cells with tRNA charging defects can induce certain adaptive response pathways that serve to restore proteostasis.^11^ Matched wild‐type (WT) and patient‐derived fibroblasts with the compound heterozygote mutations I212T/M487V in *EPRS1* were used to compare expression of EPRS1 and total protein synthesis (Figure S3), induction of ISR‐directed gene expression (Figures S4 and S6), and cell viability (Figure S5 and S7) in response to stress induction. As described in Supporting Information: Methods and Results, no significant differences were observed between WT and patient fibroblasts in these assays.

## 4 DISCUSSION

Exome sequencing of patient II:2, indicated the presence of a previously undescribed compound heterozygous variant *EPRS1* gene. The significant aminoacylation defects determined for these two EPRS1 variants support the diagnosis of our patient. The family benefitted from the functional analysis supporting the *EPRS1* mutations as the likely cause of the disease because while the diagnosis was being made, the mother I:1 was pregnant again. Prenatal testing by amniocentesis revealed that the fetus lacked the two variants and was born healthy (boy II:3).

The neurological disease‐associated *EPRS1* mutations moderately disrupt tRNA^Glu^ binding and the subtle conformational differences observed, especially for the I212T variant, likely contribute to reduced tRNA affinity and aminoacylation activity. Based on our previous studies,^4^ we expected to observe decreased cell viability of fibroblasts derived from patients encoding EPRS I212T and M487V variants. Surprisingly, this was not the case (Figure S5). Moreover, patient‐derived fibroblasts encoding EPRS1 I212T and M487V variants showed similar stress‐related gene expression as the control cells under stress conditions (Figure S4) and EPRS1 levels also appeared unchanged (Figure S3).

Our study suggests that the disease‐associated EPRS1 point mutations reported here result in significant reductions in canonical tRNA aminoacylation activity in vitro, but that these reductions are not sufficient to trigger the ISR pathway in fibroblast cells. Tissue‐specific differences in sensitivity to ARS defects have previously been observed.^12^ Proteotoxic stress in neurons is also associated with defects in the translation quality control machinery.^13^, ^14^, ^15^ Thus, it is likely that the effect of the *EPRS1* mutations would be more evident in brain tissues, which may explain the absence of cell viability differences and changes in ISR induction in mutant fibroblasts. Alternatively, a mechanism independent of global protein synthesis reduction may contribute to pathogenesis.

## AUTHOR CONTRIBUTIONS

Karin Musier‐Forsyth, Ronald C. Wek, and Vincent Michaud conceptualized the project and acquired funding, Danni Jin performed biochemical assays and analyzed data. Sheree A. Wek and Ricardo A. Cordova performed cell‐based assays and analyzed data, Didier Lacombe described clinical features of patient, Vincent Michaud performed whole exome sequencing analysis, Danni Jin, Karin Musier‐Forsyth, Ronald C. Wek and Vincent Michaud drafted the manuscript, Danni Jin, Didier Lacombe, Karin Musier‐Forsyth, Ronald C. Wek, Sheree A. Wek, and Vincent Michaud edited the manuscript.

## CONFLICT OF INTEREST

Ronald C. Wek is a member of the advisory board in HiberCell, Inc.; other authors declare no conflicts of interest.

### PEER REVIEW

The peer review history for this article is available at https://publons.com/publon/10.1111/cge.14269.

## ETHICS STATEMENT

Written informed consent was received from the patient and the participating family according to the declaration of Helsinki principles of medical research involving human subjects. Procedures were approved by the Comité de Protection des Personnes Bordeaux Outre‐Mer III.

## Supporting information


**Appendix S1.** Supporting Information

**Figure S1.** Exome sequencing results. Results show both variants in patient with one variant inherited from each parent (Alamut Visual Plus, Sophia Genetics).
**Figure S2.** Gel of purified ERS proteins, representative tRNA binding assays, and circular dichroism spectra. (A). Purified MBP‐ERS proteins analyzed on a denaturing 10% polyacrylamide gel. (B). Representative gel images of protein‐tRNA EMSAs. (C). Circular dichroism (CD) spectra and thermal melting curves for WT and mutant MBP‐ERS. Top: CD spectra collected at 20°C. For each protein, three trials were performed, and one representative spectrum is shown. Bottom: Thermal melting curves generated by monitoring CD signals at 222 nm from 20 to 90°C. Each measurement was performed in triplicate and a representative curve is shown.
**Figure S3.** Expression of EPRS1 and total protein synthesis in WT and *EPRS1* mutant fibroblasts in response to ER stress. Wild‐type (WT) and *EPRS1* mutant fibroblast cells (Mut) were treated with 1.0 μM thapsigargin (Tg, +) or vehicle for 6 h. Puromycin (1 μM) was added to culture media 15 min prior to preparation of protein lysates. Puromycin incorporation was measured by immunoblot analyses using anti‐puromycin antibody. Levels of EPRS1 and actin proteins were also shown in the immunoblot analyses. Levels of puromycin incorporation and EPRS1 protein are presented and normalized to the WT cells treated with vehicle. (A). Representative immunoblots. (B). Quantification of puromycin (top) and EPRS1 protein levels (bottom) from three biological replicates shown in (C). The top of the bar represents the mean value and *e*rror bars represent SD (*n* = 3). Statistical significance was determined using a one‐way analysis of variance (ANOVA) with Tukey's multiple comparisons. The “***” represents *p* ≤ 0.001 and “****” *p* ≤ 0.0001. Molecular weight markers are shown in kD for the immunoblot panels.
**Figure S4.** Induction of the ISR‐directed gene expression in WT and EPRS1 mutant cells during ER stress. Wild‐type (WT) and mutant (Mut) EPRS1 fibroblast cells were treated with 1 μM thapsigargin (Tg) or no treatment (NT) for 6 h. RNA was prepared from the cells and the relative levels of ATF4 and CHOP mRNAs were measured by qRT‐PCR. Data points are shown for three independent experiments and a two‐tailed Student's *t*‐test was performed to determine statistical significance. *p*‐values <0.05 are indicated by an “*”, which indicates significant differences in ATF4 mRNA levels between NT and Tg treated and between WT and Mut EPRS1 cells treated with thapsigarin. The top of the bar (black horizontal line) represents the median value.
**Figure S5.** Cell viability of WT and EPRS1 mutant cells in response to ER stress. Fibroblast cells were treated with up to 0, 0.5, 1.0, or 2.0 mM thapsigargin (Tg), as indicated, for 24, 48, or 72 h. Cell viability was measured by the MTT assay and values were normalized to WT nontreated cells and are presented as percent cell viability. Results represent six biological replicates and are shown as a bar graph. The top of the bar represents the mean value and *e*rror bars represent SD. P‐values with significant differences between wild‐type (WT) and mutant EPRS1 (Mut) cells are indicated by an “*”: *p* = 0.0004 for 0.5 μM treatment at 24 h; *p* = 0.034 for 0.5 μM and *p* = 0.0007 for 2.0 μM treatments for 48 h; and *p* = 0.0009 for 0.5 μM, *p* = 0.0001 for 1.0 μM, and *p* = 0.0007 μM treatments for 72 h.
**Figure S6.** Measurement of ISR‐directed gene expression in WT and EPRS1 mutant cells treated with GCN2iB. Wild‐type (WT) and mutant EPRS1 (Mut) fibroblast cells were treated with 2 μM GCN2iB, a potent inhibitor of GCN2 activity, or no treatment (NT) for 6 h. RNA was prepared from the cells and ATF4 and CHOP mRNAs were measured by qRT‐PCR. Data points are shown for three independent experiments and statistical analyses were carried out using a two‐tailed Student's *t*‐test. The top of the bar (black horizontal line) represents the median value.
**Figure S7.** Cell viability of WT and mutant EPRS1 cells in response to GEN2iB treatment. Wild‐type (WT) and EPRS1 mutant (Mut) fibroblast cells were treated with 0, 0.5, 1.0, or 2.0 μM GCN2iB, as indicated, for 24, 48, or 72 h. Viability of cells was measured by the MTT assay. Values were normalized to WT nontreated cells and are presented as percent cell viability. The results represent five biological replicates. Statistical analyses were carried using a two‐tailed Student's *t*‐test. The top of the bar represents the mean value and error bars represent SD.

## Data Availability

The data that support the findings of this study are available from the corresponding author upon reasonable request.
