# Supplementary material for: Aminoacylation‐defective bi‐allelic mutations in human EPRS1 associated with psychomotor developmental delay, epilepsy, and deafness
Source: Clin Genet. 2022 Dec 1;103(3):358–63. doi: 10.1111/cge.14269 (PMC9898101; doi:10.1111/cge.14269)
Supplement: Supplementary file 1 — Appendix S1. Supporting Information [file CGE-103-358-s001.docx]

**Supporting information: Methods and Results**

Aminoacylation-defective bi-allelic mutations in human EPRS1 associated with psychomotor developmental delay, epilepsy, and deafness

### Methods

### Protein expression and purification

Small ubiquitin-like modifier (SUMO)- and maltose-binding protein (MBP)-tagged ERS proteins containing aa. 1-749 of human EPRS were expressed and purified as previously described ^1^. In brief, expression of MBP-ERS in *Escherichia coli* BL21(DE3)RIL was induced with 0.1 mM isopropyl β-D-1-thiogalactopyranoside (IPTG) at 16° C overnight. Bacterial lysate was treated with 0.5% v/v polyethyleneimine (PEI) for nucleic acid removal and proteins were precipitated with 375 mg/mL ammonium sulfate. The recovered proteins were first purified using His-Select Nickel affinity chromatography, subjected to SUMO protease (Sigma-Aldrich) digestion for SUMO tag cleavage, and further purified through size-exclusion chromatography using a HiLoad Superdex 200 16/60 column (GE Healthcare). Proteins were concentrated and stored at -20 °C in 12.5 mM Tris-HCl pH 7.5, 75 mM NaCl, 0.5 mM dithiothreitol (DTT) and 40% glycerol. Protein concentration was measured using the Pierce BCA protein assay kit (Thermo Fisher).

### *In vitro* transcription and labeling of human tRNA^Glu^

Human tRNA^Glu(TTC)^ was prepared through T7 RNA polymerase *in vitro* transcription as described ^1,2^ and the 3'-adenosine of the tRNA was labeled with ^32^P ^3^. Prior to assays, tRNA was refolded by diluting to approximately 15 μM in 20 mM Tris-HCl pH 7.5, incubating at 80 °C for 2 min, 60 °C for 2 min, followed by adding 10 mM MgCl_2_ and incubation at room temperature for 5 min. For 3'-[^32^P] labeling, the folded RNA was incubated with 25 μL α-[^32^P] ATP (PerkinElmer), 10 mM MgCl_2_, 50 μM sodium pyrophosphate, 50 mM glycine and 0.1 mg/mL nucleotidyltransferase for 5 min at 37 °C. The reaction was then incubated for 2 min at 37 °C in the presence of CTP (1 μM) and pyrophosphatase (10 units/mL). The labeled RNA was subjected to acidic phenol/chloroform extraction followed by Sephadex G-25 column purification (Roche) to remove free ATP. Purified RNA was ethanol-precipitated, re-dissolved in water and stored at -80 °C.

### Aminoacylation assays

Two types of aminoacylation assays were performed in this study. For the assays using sub-saturating amino acid, reactions were performed with 20 mM Tris-HCl pH 7.5, 20 mM KCl, 10 mM MgCl_2_, 0.1 mg/mL bovine serum albumin (BSA), 4 mM DTT, 4 mM ATP, 20 μM glutamic acid, 0.3 μCi/μL [^3^H]-glutamic acid and 0.1 μM unlabeled tRNA^Glu^ at 37 °C. Aminoacylation reactions were initiated by adding MBP-ERS proteins to a final concentration of 4 nM (for WT MBP-ERS) or 10 nM (for I212T and M487V MBP-ERS). Reactions were quenched onto Whatman 3MM filter paper pre-spotted with 5% trichloroacetic acid (TCA). The filter pads were washed three times with 5% TCA and one time with 95% ethanol, air-dried and subjected to scintillation counting using a Beckman Coulter LS6500 scintillation counter. Assays were performed in triplicate and the catalytic efficiency, *k*_cat_/*K*_M_, was estimated based on the initial reaction rate.

An alternative method to measure aminoacylation activity used [^32^P]-labeled tRNA and saturating levels of unlabeled glutamic acid ^4,5^. The reaction mix contained 1 mM glutamic acid, 0.5 μM 3'-[^32^P]-tRNA^Glu^ and all other reaction buffer components described above. Reactions were initiated by the addition of 10 nM WT or mutant MBP-ERS. At selected time points, 2 μL aliquots were quenched into a solution containing 200 mM sodium acetate pH 5.0, approximately 2 unit/uL S1 nuclease and 1x S1 nuclease reaction buffer (Promega) followed by a 30-min incubation at room temperature. Reaction products were separated on a polyethylenimine (PEI)-cellulose thin-layer chromatography (TLC) plate (Sigma) using a running buffer containing 100 mM ammonium acetate and 5% acetic acid. Results were quantified by phosphorimaging using a Typhoon Scanner (GE Healthcare) and ImageQuant software. Each assay was performed in quadruplicate and the catalytic efficiency, *k*_cat_/*K*_M_, was estimated based on the initial reaction rate.

### Electromobility gel shift assays (EMSAs)

EMSAs were performed with 10 nM [^32^P]-labeled tRNA and serially diluted MBP-ERS recombinant proteins in the presence of 25 mM Tris-HCl pH 7.5, 50 mM NaCl, 1 mM MgCl_2_, 1 mM DTT and 5 μg/mL heparin. Reactions were initiated by adding WT or mutant MBP-ERS (0 to 10 μM) into the reaction mix containing tRNA. Reactions were incubated at room temperature for 30 min, mixed with 6x loading dye [50% glycerol in Tris-Borate (TB) buffer containing xylene cyanol and bromophenol blue], and run on pre-cast native 4-16% polyacrylamide mini protein gels (ThermoFisher) on ice. Running buffer was 1x TB containing 1 mM MgCl_2_. Results were quantified by phosphorimaging as described above. Each assay was performed in triplicate and the dissociation constant (*K*_d_) was determined by plotting fraction bound vs. protein concentration and fitting the data to the Hill equation as described.^6^

### Circular dichroism (CD) spectroscopy

For CD experiments, 0.5 mg/mL MBP-ERS (WT and mutants) in 50 mM sodium phosphate, pH 7.8, was loaded into 1 mm pathlength quartz cuvettes. CD spectra were collected from 200 to 275 nm at 20 °C using a Jasco J-815 CD spectrometer. Thermal melting curves were collected by monitoring the CD signal at 222 nm over a 20 – 90 °C temperature range. Melting data were fit to an adapted Hill equation to derive apparent melting temperature, T_m_, as previously described ^1^.

### Limited protease digestion assays

Trypsin (Proti-Ace™ Kit, Hampton Research HR2-429) was reconstituted according to the manufacturer’s protocol and diluted to 4 μg/mL with reaction buffer (10 mM HEPES pH 7.5, 500 mM NaCl). To start the reaction, 4 mg/mL MBP-ERS in storage buffer (12.5 mM Tris HCl pH 7.5, 75 mM NaCl, 0.5 mM DTT and 40% glycerol) was mixed with an equal volume of 4 μg/mL trypsin. Reactions were incubated at 37 °C and quenched at desired time points into denaturing gel protein loading buffer. For control reactions, proteins were mixed with reaction buffer and incubated at 37 °C. Samples were analyzed on SDS-10% polyacrylamide gels.

**Cell culture, immunoblots, and qRT-PCR**

The patient-derived mutant and control wild-type fibroblasts were cultured in DMEM supplemented with 10% FBS to about 70% confluency and then treated with 1 μM thapsigargin or no stress treatment for 6 h. Cells were collected, and protein lysates were prepared and separated by SDS-PAGE. Immunoblot analyses were carried out using the following antibodies: EPRS1 (Abcam ab31531) and β-actin (Sigma-Aldrich A5441) and antibody-bound proteins were imaged using chemiluminescence and a ChemiDoc Imaging System (BioRad). RNA was prepared from the wild-type and the patient-derived cells cultured in the presence of 1 μM thapsigargin, 2 μM GCN2iB, or no stress treatment for 6 h, as indicated. Relative levels of *ATF4* and *CHOP* mRNAs were measured by real-time quantitative reverse transcription PCR (qRT-PCR) with the following primers: CHOP (forward: 5'- AGCCAAAATCAGAGCTGGAA-3', reverse: 5'- ACAAGTTGGCAAGCTGGTCT-3'); ATF4 (forward: 5'- TCAAACCTCATGGGTTCTCC -3', reverse: 5'-GTGTCATCCAACGTGGTCAG-3'); and for normalization Actin (forward: 5'- GGACTTCGAGCAAGAGATGG-3', reverse: 5'-AGCACTGTGTTGGCGTACAG-3-). Data was derived from 3 biological replicates and analyzed using a two-tailed student t-test, error bars represent S.D.

**MTT Assays**

Equal amounts of wild-type and *EPRS1* mutant fibroblast cells were cultured in DMEM supplemented with 10% FBS in 96-well culture plates at 5,000 cells/well. The following day, cells were treated with up to 0.5, 1.0, or 2.0 μM thapsigargin for up to 72 h or no stress treatment, as indicated. Alternatively, the cells were exposed to 0.5, 1.0, or 2.0 μM GCN2iB for up to 72 h or no stress treatment, as indicated. Cell viability was evaluated by the conversion of tetrazolium (MTT) to formazan via measuring the absorbance at 570 nm using the CellTiter 96-well nonradioactive cell proliferation assay (Promega, #G4000). Absorbance values were normalized to non-stress wild-type cells and presented as cell viability. A one-way ANNOVA was carried out for statistical analyses and error bars are S.D; n=5.

**Protein synthesis measurements**

To measure total mRNA translation, 1 µM puromycin (MP Biomedicals Cat #194539) was included in the culture medium for the last 15 min of treatment with 1 µM thapsigargin for 6 h or vehicle. Protein lysates were prepared and probed and quantified by immunoblot analyses using anti-puromycin antibody (Millipore Cat. #MABE343, RRID:AB_2566826), or with measurements of EPRS1 and actin proteins, as described above.

**Whole-exome sequencing**

Library preparation, exome capture, sequencing and data analysis were performed by IntegraGen SA (Evry, France) using Twist Bioscience in-solution enrichment methodology (Agilent, Santa Clara, California), followed by paired-end 100 base massively parallel sequencing on Illumina NovaSeq (Illumina, San Diego, California). Image analysis and base calling was done by Illumina Real Time Analysis software with default parameters. Reads were aligned to reference sequence (GRCh38) by Burrows-Wheeler Aligner (BWA). Duplicates were removed. Variant calling was done using GATK Haplotype Caller GVCF (3.7) [quality cut off: Q(SNPs) < 10, Q(Indel) < 20, QVCutoff <90)]. Quality control (QC) was performed on the resulting FASTQ (FastQC). Coverage of index case was above 25X for 98% of the targeted region. Variant annotation and filtering were performed with Alissa Interpret (Agilent) using trio filtering strategy. Parameters included frequency below 1% in population databases (gnomAD), variants described in HGMD or Clinvar, truncating variants or splice variants, mode of inheritance. Visualization of BAMs was done with Alamut Visual Plus (Sophia Genetics). Pathogenic variants were confirmed with Sanger trio sequencing (BDT v3.1 on ABI3500xL Dx). Both *EPRS1* variants were uploaded to the Clinvar database (SCV002562256 for variant c.1459A>G and SCV002562205 for variant c.635T>C).

**Results**

**Clinical Results**

Both *EPRS1* variants (NM_004446.3:c.1459A>G/p.Met487Val and NM_004446.3:c.635T>C/p.Ile212Thr) were classified as pathogenic as they meet American College of Medical Genetics and Genomics (ACMG) criteria PS3 (well-established *in vitro* or *in vivo* functional studies supportive of a damaging effect on the gene or gene product), PM2 (absent from controls in gnomAD), PM3 (for recessive disorders, detected in *trans* with a pathogenic variant), and PP3 (multiple lines of computational evidence support a deleterious effect on the gene or gene product).

**Computational Predictions**

According to the PolyPhen-2 server,^7^ both I212T and M487V EPRS1 substitutions were predicted as probably damaging (HumDiv model) or possibly damaging (HumVar model). DynaMut predictions,^8^ which are based on the Alphafold-predicted EPRS1 protein structure, indicated that both substitutions result in a gain of protein flexibility. M487V has a mild, local effect on EPRS1 stability (ΔΔG=-0.987 kcal/mol), whereas I212T has a stronger, more global destabilizing effect (ΔΔG=3.469 kcal/mol), consistent with the observation that I212T variant is more susceptible to limited protease digestion compared to the WT or M487V variant (Figure 3C).

**Cell-based Assays**

The integrated stress response (ISR) induces the transcription and translation of *ATF4* and *CHOP*, which direct expression of genes that serve to restore proteostasis.^9^ While the ISR enables cells to adapt to stress and restore protein homeostasis, chronic induction of the ISR can instead cause cell death.^10,11^ We wished to determine whether there is altered induction of the ISR in cells expressing the mutant EPRS1. Matched wild-type (WT) and patient-derived fibroblasts with the compound heterozygote mutations I212T/M487V in *EPRS1* were treated with thapsigargin, a well characterized inducer of endoplasmic reticulum (ER) stress. There were similar levels of EPRS1 protein in the WT and mutant cells and amounts were not appreciably changed in response to stress (Figure S3). Total protein synthesis was evaluated using a puromycin incorporation assay. There was no statistically significant difference in newly synthesized protein between WT and mutant cells, either in the presence or absence of thapsigargin (Figure S3).

There was a potent increase in *ATF4* and *CHOP* mRNA in both the WT and mutant fibroblasts subjected to the ER stress, albeit the cells expressing the mutant EPRS1 showed a modest, but significant, lowering in induction of *ATF4* (Figure S4). Induction of *CHOP* mRNA in WT and mutant cells showed no significant difference between the WT and EPRS1 mutant cells. Furthermore, viability as measured by the MTT assay was similarly lowered in both WT and mutant fibroblasts treated with upwards of 2 mM thapsigargin for 24h, while mutant cells showed a modest but significantly higher viability than WT cells upon 48 h and 72 h of thapsigargin treatment (Figure S5).

In the ISR, the sensor protein GCN2 protein can be activated by deficient aminoacylation of tRNAs, which helps restore proteostasis and provides for cell protection.^9,12,13^ We considered the idea that intermittent changes in tRNA^Glu^ charging and GCN2 activity may occur in mutant fibroblasts. In this case inhibition of GCN2 would possibly disrupt basal ISR induction and cell viability. We therefore treated the WT and mutant fibroblast cells with GCN2iB, a potent inhibitor of GCN2 activity ^14^ and measured induction of ISR expression and cell viability. GCN2iB did not significant affect the expression of *ATF4* and *CHOP* mRNA, with similar levels independent of EPRS1 function and GCN2iB treatment (Figure S6). Furthermore, cell viability was largely unaffected by treatment with up to 2 μM GCN2iB for 24, 28, or 72 hours (Figure S7). As noted above, cells expressing mutant *EPRS1* demonstrated increased survival upon thapsigargin treatment relative to WT cells, suggesting that these genetic changes provided protection against acute ER stress (Figure S5). Collectively, these results suggest that despite the enzymatic changes observed in the EPRS1 variants, we observed minimal effects in ISR expression and cell viability in cultured fibroblasts.

## References

1. Jin D, Wek SA, Kudlapur NT, et al. Disease-associated mutations in a bifunctional aminoacyl-tRNA synthetase gene elicit the integrated stress response. *J Biol Chem*. 2021;297(4):101203.

2. Milligan JF, Uhlenbeck OC. Synthesis of small RNAs using T7 RNA polymerase. *RNA Process Part A Gen Methods*. 1989;180:51-62.

3. Ledoux S, Uhlenbeck OC. [3’-^32^P]-labeling tRNA with nucleotidyltransferase for assaying aminoacylation and peptide bond formation. *Methods*. 2008;44(2):74-80.

4. Wolfson AD, Pleiss JA, Uhlenbeck OC. A new assay for tRNA aminoacylation kinetics. *RNA*. 1998;4(8):1019-1023.

5. Wolfson AD, Uhlenbeck OC. Modulation of tRNA^Ala^ identity by inorganic pyrophosphatase. *Proc Natl Acad Sci U S A*. 2002;99(9):5965-5970.

6. Ryder SP, Recht MI, Williamson JR. Quantitative Analysis of Protein-RNA Interactions by Gel Mobility Shift. In: Lin R-J, ed. *RNA-Protein Interaction Protocols*. Humana Press; 2008:99-115.

7. Adzhubei IA, Schmidt S, Peshkin L, et al. A method and server for predicting damaging missense mutations. *Nat Methods*. 2010;7(4):248-249.

8. Rodrigues CHM, Pires DE V, Ascher DB. DynaMut: predicting the impact of mutations on protein conformation, flexibility and stability. *Nucleic Acids Res*. 2018;46(W1):W350-W355.

9. Wek RC. Role of eIF2α Kinases in Translational Control and Adaptation to Cellular Stress. *Cold Spring Harb Perspect Biol*. 2018;10(7):a032870. doi:10.1101/cshperspect.a032870

10. Marciniak SJ, Yun CY, Oyadomari S, et al. CHOP induces death by promoting protein synthesis and oxidation in the stressed endoplasmic reticulum. *Genes Dev* . 2004;18(24):3066-3077.

11. Tabas I, Ron D. Integrating the mechanisms of apoptosis induced by endoplasmic reticulum stress. *Nat Cell Biol*. 2011;13(3):184-190. doi:10.1038/ncb0311-184

12. Ishimura R, Nagy G, Dotu I, Chuang JH, Ackerman SL. Activation of GCN2 kinase by ribosome stalling links translation elongation with translation initiation. *Elife*. 2016;5:e14295.

13. Misra J, Holmes MJ, T. Mirek E, et al. Discordant regulation of eIF2 kinase GCN2 and mTORC1 during nutrient stress. *Nucleic Acids Res*. 2021;49(10):5726-5742.

14. Nakamura A, Nambu T, Ebara S, et al. Inhibition of GCN2 sensitizes ASNS-low cancer cells to asparaginase by disrupting the amino acid response. *Proc Natl Acad Sci*. 2018;115(33):E7776-E7785.
