# Supplementary material for: Aminoacylation‐defective bi‐allelic mutations in human EPRS1 associated with psychomotor developmental delay, epilepsy, and deafness
Source: Clin Genet. 2022 Dec 1;103(3):358–63. doi: 10.1111/cge.14269 (PMC9898101; doi:10.1111/cge.14269)
Supplement: Supplementary file 2 — Figure S1. Exome sequencing results. Results show both variants in patient with one variant inherited from each parent (Alamut Visual Plus, Sophia Genetics). Figure S2. Gel of purified ERS proteins, representative tRNA binding assays, and circular dichroism spectra. (A). Purified MBP‐ERS proteins analyzed on a denaturing 10% polyacrylamide gel. (B). Representative gel images of protein‐tRNA EMSAs. (C). Circular dichroism (CD) spectra and thermal melting curves for WT and mutant MBP‐ERS. Top: CD spectra collected at 20°C. For each protein, three trials were performed, and one representative spectrum is shown. Bottom: Thermal melting curves generated by monitoring CD signals at 222 nm from 20 to 90°C. Each measurement was performed in triplicate and a representative curve is shown. Figure S3. Expression of EPRS1 and total protein synthesis in WT and EPRS1 mutant fibroblasts in response to ER stress. Wild‐type (WT) and EPRS1 mutant fibroblast cells (Mut) were treated with 1.0 μM thapsigargin (Tg, +) or vehicle for 6 h. Puromycin (1 μM) was added to culture media 15 min prior to preparation of protein lysates. Puromycin incorporation was measured by immunoblot analyses using anti‐puromycin antibody. Levels of EPRS1 and actin proteins were also shown in the immunoblot analyses. Levels of puromycin incorporation and EPRS1 protein are presented and normalized to the WT cells treated with vehicle. (A). Representative immunoblots. (B). Quantification of puromycin (top) and EPRS1 protein levels (bottom) from three biological replicates shown in (C). The top of the bar represents the mean value and error bars represent SD (n = 3). Statistical significance was determined using a one‐way analysis of variance (ANOVA) with Tukey's multiple comparisons. The “***” represents p ≤ 0.001 and “****” p ≤ 0.0001. Molecular weight markers are shown in kD for the immunoblot panels. Figure S4. Induction of the ISR‐directed gene expression in WT and EPRS1 mutant cells during ER stress. Wild‐t [file CGE-103-358-s002.docx]

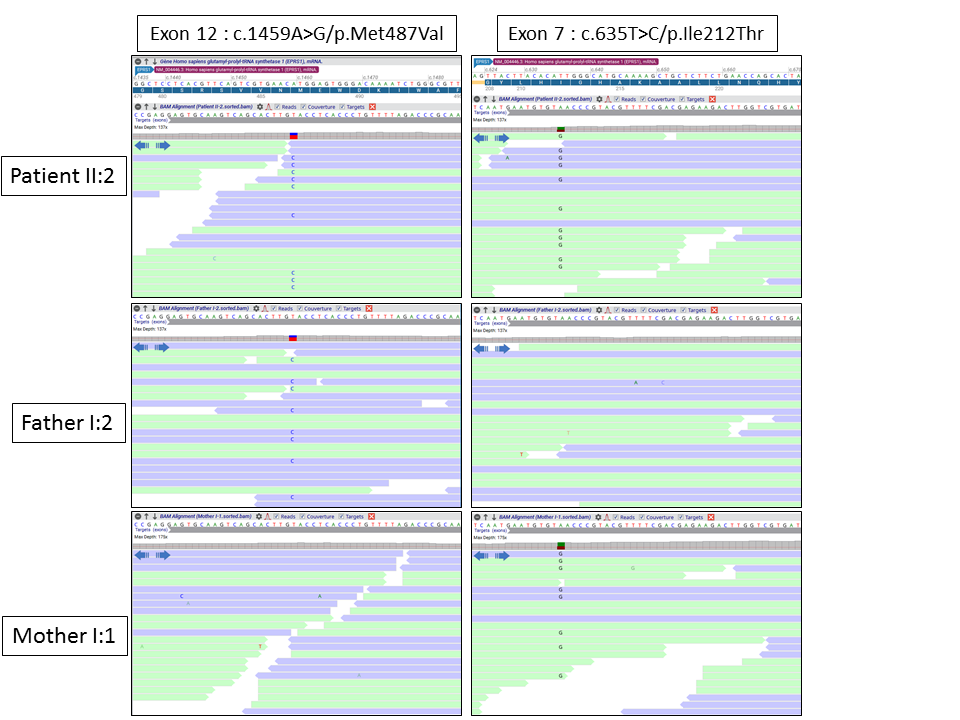


**Figure S1. Exome sequencing results**. Results show both variants in patient with one variant inherited from each parent (Alamut Visual Plus, Sophia Genetics).


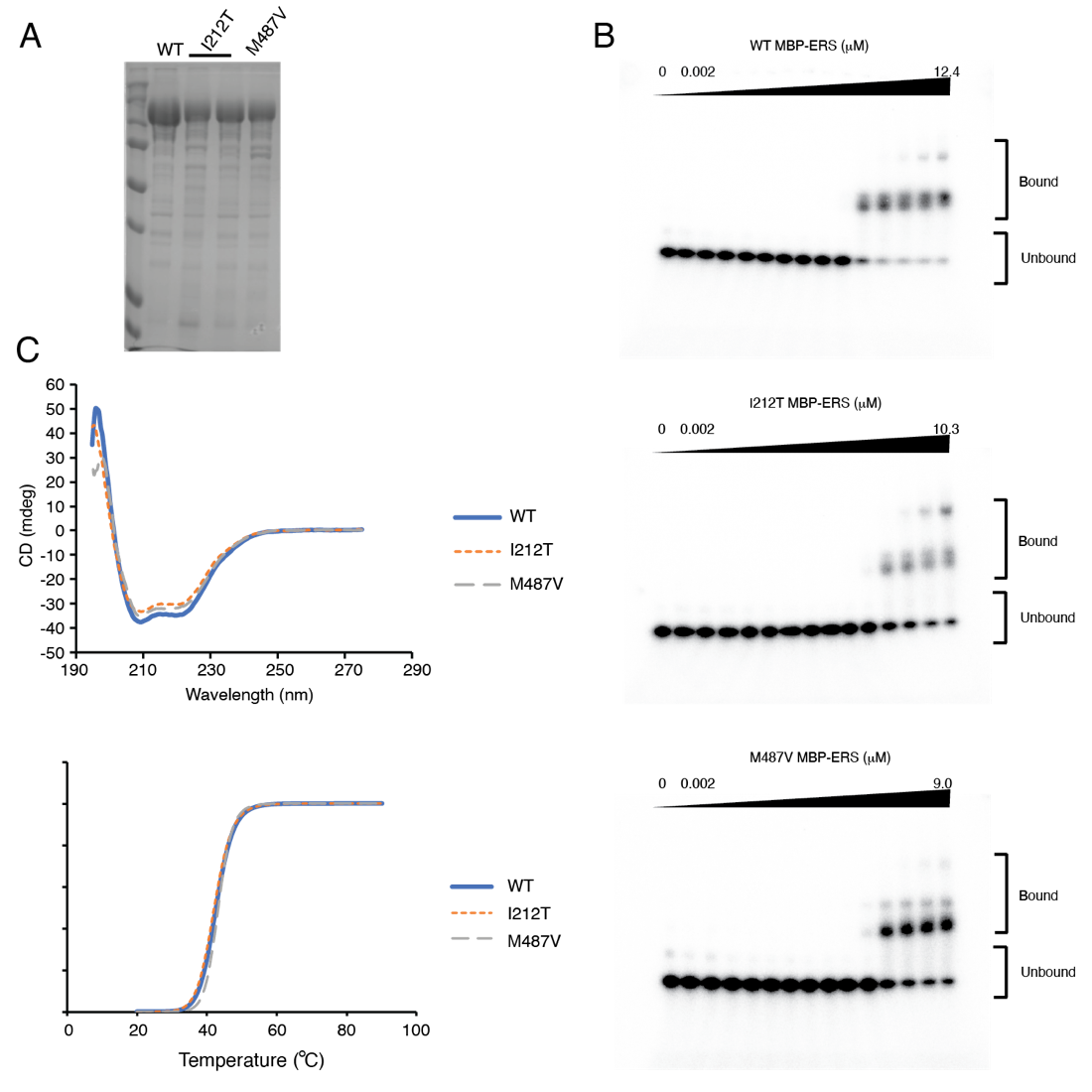


**Figure S2. Gel of purified ERS proteins, representative tRNA binding assays, and circular dichroism spectra.** (A). Purified MBP-ERS proteins analyzed on a denaturing 10% polyacrylamide gel. (B). Representative gel images of protein-tRNA EMSAs. (C). Circular dichroism (CD) spectra and thermal melting curves for WT and mutant MBP-ERS. Top: CD spectra collected at 20 °C. For each protein, 3 trials were performed, and one representative spectrum is shown. Bottom: Thermal melting curves generated by monitoring CD signals at 222 nm from 20 °C to 90 °C. Each measurement was performed in triplicate and a representative curve is shown.


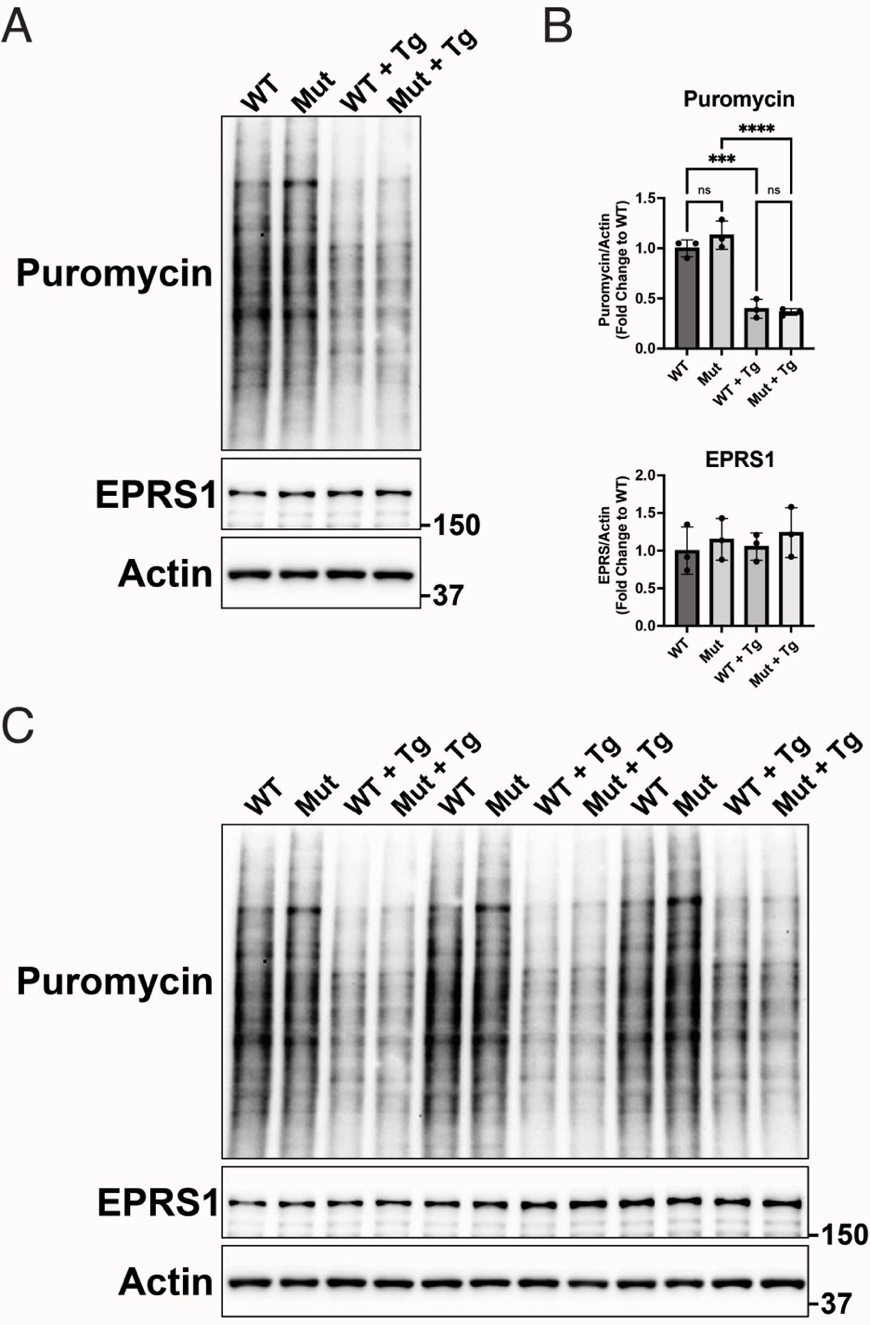


**Figure S3. Expression of EPRS1 and total protein synthesis in WT and *EPRS1* mutant fibroblasts in response to ER stress.** Wild-type (WT) and *EPRS1* mutant fibroblast cells (Mut) were treated with 1.0 μM thapsigargin (Tg, +) or vehicle for 6 h. Puromycin (1 µM) was added to culture media 15 min prior to preparation of protein lysates. Puromycin incorporation was measured by immunoblot analyses using anti-puromycin antibody. Levels of EPRS1 and actin proteins were also shown in the immunoblot analyses. Levels of puromycin incorporation and EPRS1 protein are presented and normalized to the WT cells treated with vehicle. (A). Representative immunoblots. (B). Quantification of puromycin (top) and EPRS1 protein levels (bottom) from three biological replicates shown in (C). The top of the bar represents the mean value and *e*rror bars represent SD (n=3). Statistical significance was determined using a one-way analysis of variance (ANOVA) with Tukey’s multiple comparisons. The "***" represents p ≤ 0.001 and "****" p ≤ 0.0001. Molecular weight markers are shown in kD for the immunoblot panels.


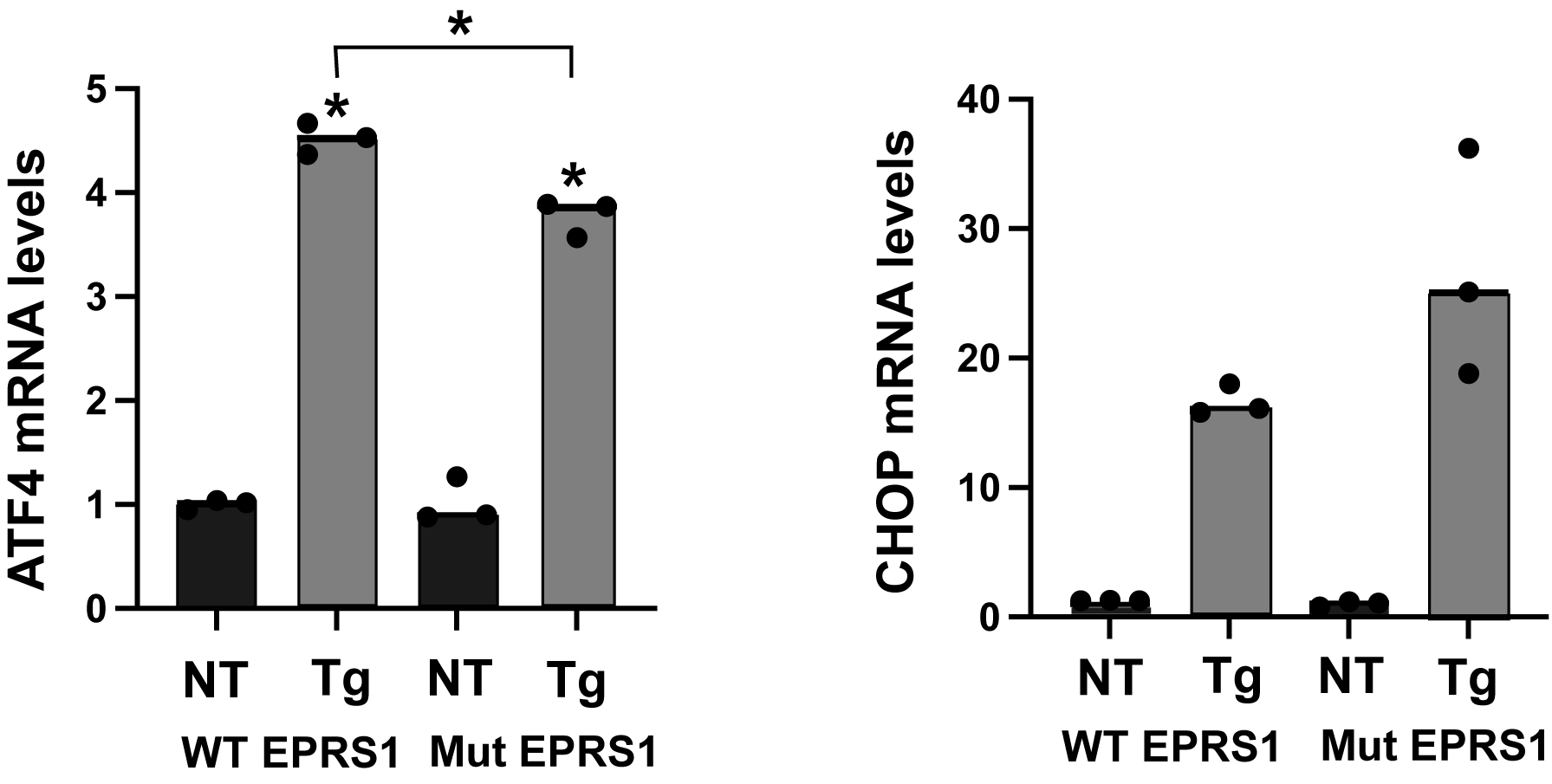


**Figure S4.** **Induction of the ISR-directed gene expression in WT and EPRS1 mutant cells during ER stress.** Wild-type (WT) and mutant (Mut) EPRS1 fibroblast cells were treated with 1 μM thapsigargin (Tg) or no treatment (NT) for 6 h. RNA was prepared from the cells and the relative levels of ATF4 and CHOP mRNAs were measured by qRT-PCR. Data points are shown for three independent experiments and a two-tailed Student's t-test was performed to determine statistical significance. P-values <0.05 are indicated by an "*", which indicates significant differences in ATF4 mRNA levels between NT and Tg treated and between WT and Mut EPRS1 cells treated with thapsigarin. The top of the bar (black horizontal line) represents the median value.


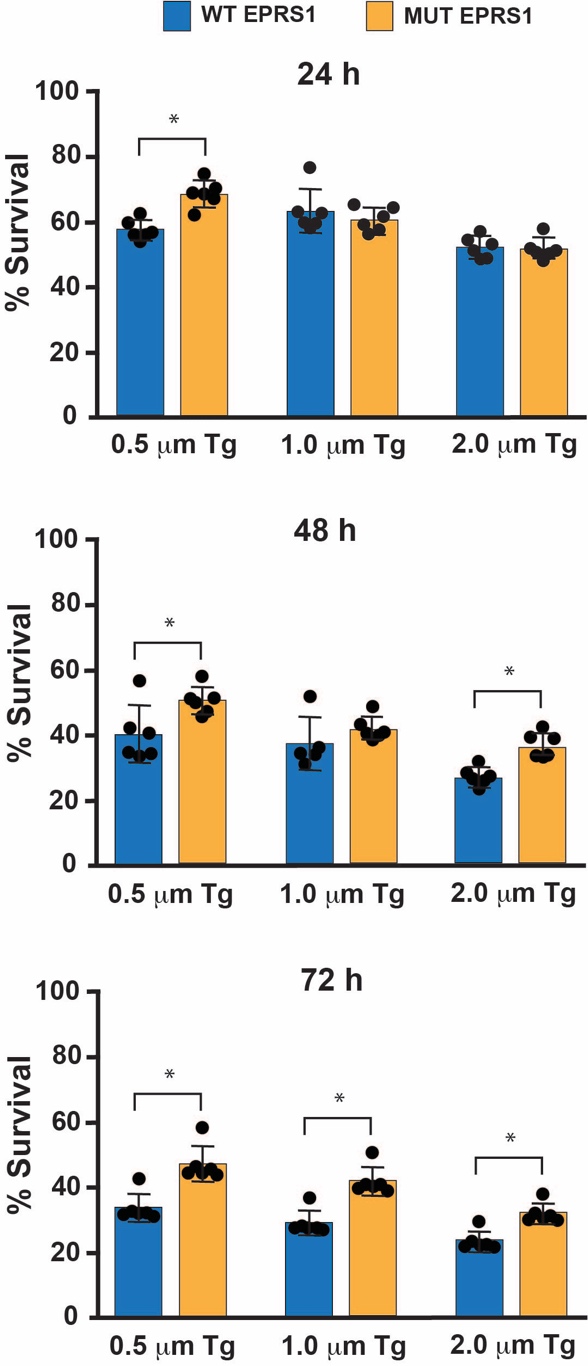


**Figure S5.** **Cell viability of WT and EPRS1 mutant cells in response to ER stress.** Fibroblast cells were treated with up to 0, 0.5, 1.0, or 2.0 mM thapsigargin (Tg*)*, as indicated, for 24, 48, or 72 h. Cell viability was measured by the MTT assay and values were normalized to WT non-treated cells and are presented as percent cell viability.

Results represent six biological replicates and are shown as a bar graph. The top of the bar represents the mean value and *e*rror bars represent SD. P-values with significant differences between wild-type (WT) and mutant EPRS1 (Mut) cells are indicated by an "*": p=0.0004 for 0.5 μM treatment at 24 h; p=0.034 for 0.5 μM and p=0.0007 for 2.0 μM treatments for 48 h; and p=0.0009 for 0.5 μM, p=0.0001 for 1.0 μM, and p=0.0007 μM treatments for 72 h.


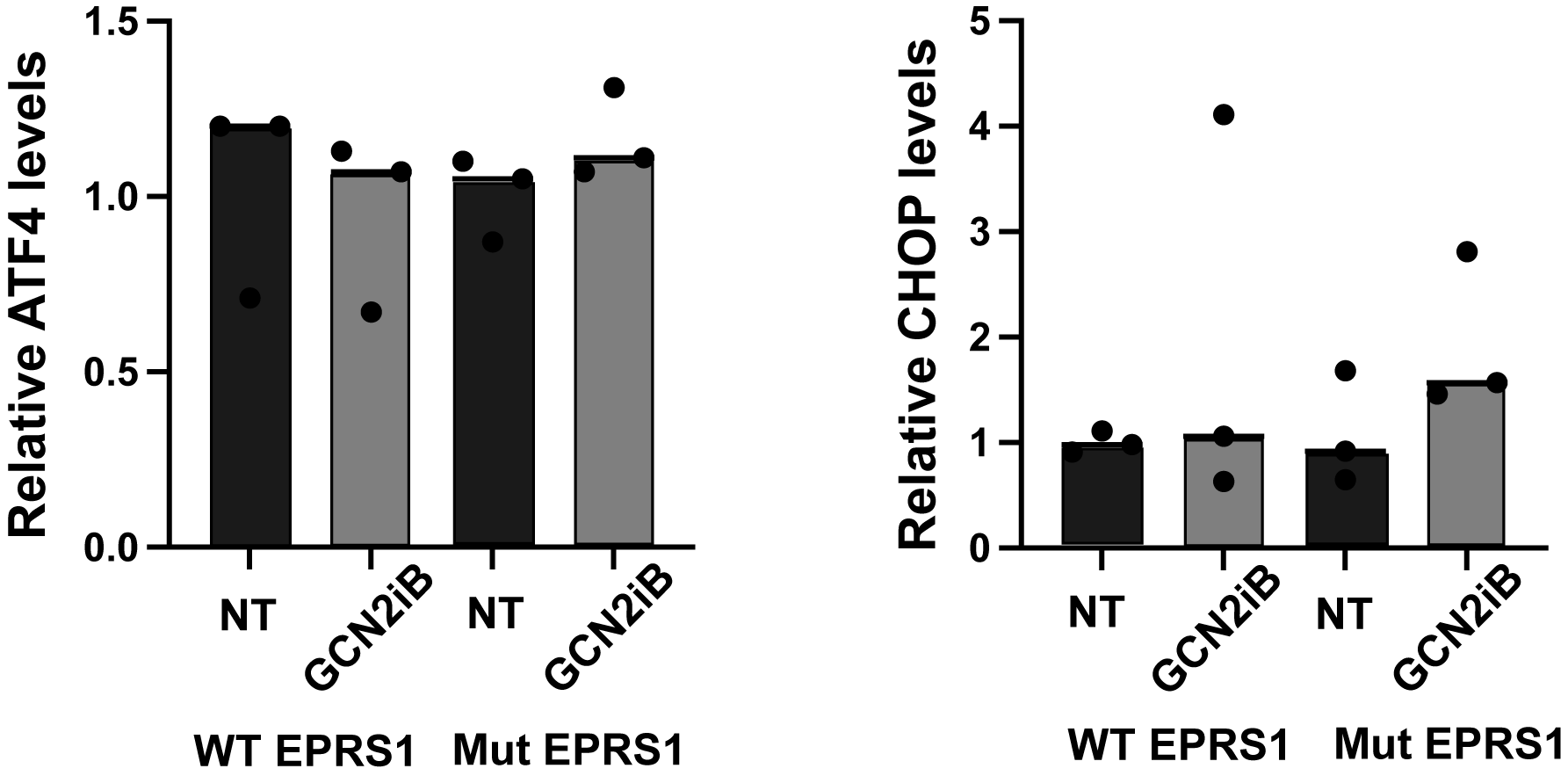


**Figure S6.** **Measurement of ISR-directed gene expression in WT and EPRS1 mutant cells treated with GCN2iB.** Wild-type (WT) and mutant EPRS1 (Mut) fibroblast cells were treated with 2 μM GCN2iB, a potent inhibitor of GCN2 activity, or no treatment (NT) for 6 h. RNA was prepared from the cells and ATF4 and CHOP mRNAs were measured by qRT-PCR. Data points are shown for three independent experiments and statistical analyses were carried out using a two-tailed Student's t-test. The top of the bar (black horizontal line) represents the median value.


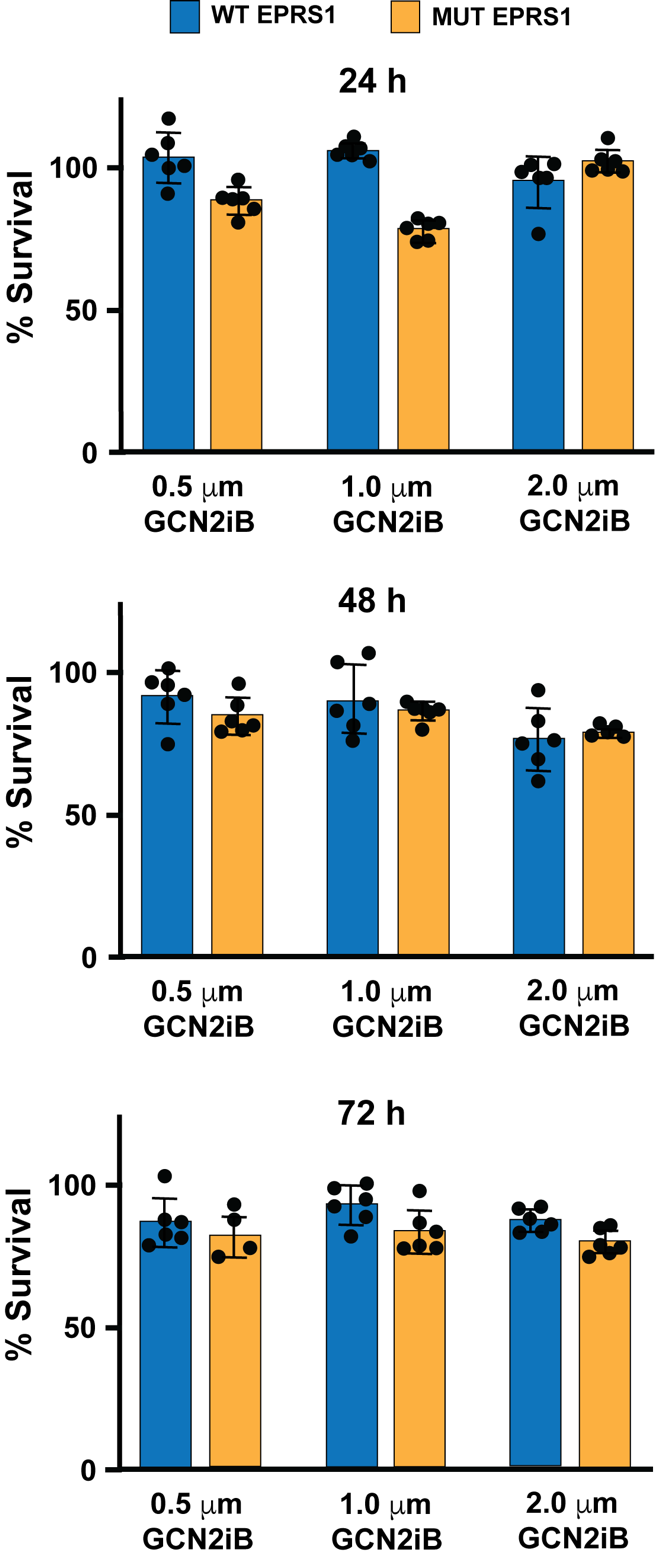


**Figure S7.** **Cell viability of WT and mutant EPRS1 cells in response to GEN2iB treatment.** Wild-type (WT) and EPRS1 mutant (Mut) fibroblast cells were treated with 0, 0.5, 1.0, or 2.0 μM GCN2iB, as indicated, for 24, 48, or 72 h. Viability of cells was measured by the MTT assay. Values were normalized to WT non-treated cells and are presented as percent cell viability. The results represent five biological replicates. Statistical analyses were carried using a two-tailed Student’s t-test. The top of the bar represents the mean value and error bars represent SD.
